# A case of ileal caecum intussusception mimicking rectal prolapse in a 4-month-old female infant

**DOI:** 10.1016/j.ijscr.2024.109572

**Published:** 2024-03-22

**Authors:** Fred Laizer, Frank Joachim, Anthony Lobulu

**Affiliations:** Department of General Surgery, Mt Meru Regional Referral Hospital, P.O Box 3092, Arusha, Tanzania

**Keywords:** Intussusception, Ilia caecum, Rectal prolapse

## Abstract

**Introduction and importance:**

Ilea caecum Intussusception protruding to the level of anus is a rare manifestation and potentially serious condition in infants.

**Case presentation:**

A four-month-old infant presented with a one-day history of non-projectile vomiting, three episodes, food contents, worsened by feeding, accompanied by intermittent low-grade fever, and one instance of passing black tarry stool. After outpatient treatment, the infant showed improvement for three days, but later the mother noticed a protruding, self-reducing anal mass, hence the suspected rectal prolapse, which was then Referred for further management.

**Clinical discussion:**

Intussusception, the most frequent surgical emergency in infants and young children aged 3 to 6 months, is primarily idiopathic, with the ileocecal region being the most commonly affected (90 % of cases). However, when the intussusceptum advances to the anus, it's rare, often leading to misdiagnosis and mismanagement.

**Conclusion:**

Intussusception of the colon should be added to the differential diagnosis of symptoms and the clinical picture of rectal prolapse.

## Highlights

• Ilea caecum intussusception advanced to the rectum is something to think out when encounter any infant with protruding mass par anus
• Atypical presentation of ilea caecum intussusception is missing leading diagnosis hence clinician should be very suspicious when dealing with infant with GI conditions

## 1 Introduction

A common abdominal condition in newborns and early children aged between 3 and 6 months is intussusception, which is the telescoping or invagination of one section of the intestine into another [1]. Rarely do instances resemble rectal prolapse, although abdominal pain, vomiting, and bloody stools are common initial symptoms [2].

The etiology of intussusception is mainly idiopathic in children; however, in some cases it is related to viral infection, notable rotaviral leading to hyperplasia of intentional mesentery Payers Parches commonly on the distal ilea, which acts as the leading [3].

The management of intussusception depends on the clinical status of the patient [4], but usually starts with initial resuscitation to a definitive managements, which is either non-surgical (such as hydrostatic enema/saline or pneumatic air) or surgical (laparoscopic or laparotomy) [5].

This work has been reported in line with the SCARE criteria [12].

### 1.1 Case report

A four-month-old female child presented with five day's history of non-projectile vomiting, three episodes of food contents vomitus per day, aggravated by feeding. This condition was accompanied by on and off lower grade fever and one episode of passing black tarry stool. She was prescribed ampiclox syrup 5mls po 8hrly for five days and paracetamol syrup 3mls po 8hrly for five days, at a nearby hospital as an outpatient. The child's health was reported to be in good progress for about three days. Over the course of the illness, the mother noted a protruding, self-reducing mass per anal, non-painful, not bleeding, no vomiting, and no abdominal distention associated with passing a normal soft stool, rectal prolapse diagnosis was reached, and she was referred to our center for father management.

On physical examination; the child was alert and active, not pale, not dehydrated and afebrile.

Vital signs; blood pressure = 95/45, pulse pate = 110bmn, respiratory rate = 45 cpm Spo2 = 98 % on room air and body temperature = 36 °C.

Per-Abdominal Examination; not distended, soft abdomen, no tenderness, palpable mobile, free mass on the left lower quadrant which was mobile and non-tender.

**Fig. 1:**
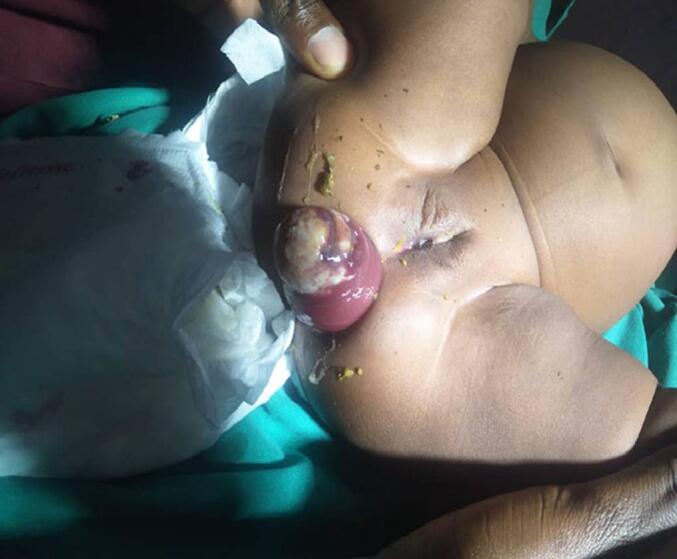
A protruding mass per anal.

Digital Rectal Examination; an obvious circumferential protruding mass per anal on straining, which reduces spontaneously; no other lesion is seen. Fig. 1.

Plane abdominal x ray done shows nothing significant.

Abd ultrasound findings were specific to features of intussusception, including the “target sign,” with a remarkable end and suggestive features of edematous bowel.

Electrolyte results revealed mild hyponatremia of 133.01 mmol/l, with normal chlorine and potassium levels, CBC was essentially normal paramitas, with Hb 11.3 g/dl.

**Fig. 2:**
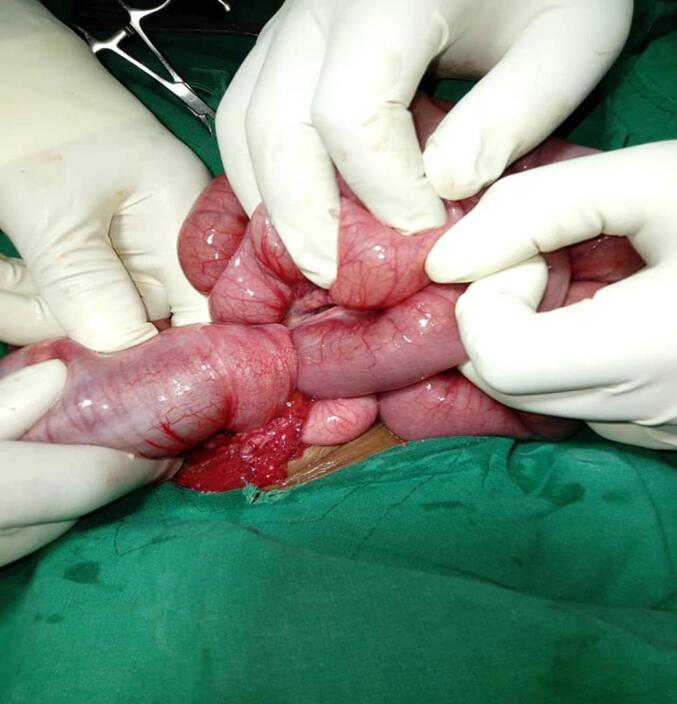
Intraoperative ileal cecal intussusception.

The diagnosis of intussusception was based on the history, clinical examination, laboratory, and radiological results. Plan for emergency laparotomy procedure reached, in which intraoperative finding was ilea caecum intussusception, which advanced to the anus with edematous bowel (Fig. 2), milking was managed only up to the level of transverse colon, then it was impossible to progress up to the caecum due to oedema, hence right hemicolectomy was done and ilea transverse end-end anastomosis performed. The child was admitted to the pediatric ICU, whereby on the 2nd day, the child was doing well and transferred to a general ward, started to feed and pass stool normally. On the 4th day the patient was discharged home, and with a follow up on surgical outpatients' clinic for six weeks, with no complications were reported.

## 2 Discussion

Intussusception is the commonest surgical emergency affecting infant and children mainly at an age of 3 months to 6 months [6] with estimated mortality of 9.4 % [7].

Ileal caecum intussusception is common reported type 90 % of cases [2], the classic presentation of intussusception is colicky abdominal pain, vomiting and passing black carry stool [8], however in some cases may have atypical presentation which often mislead clinicians to diagnose, hence delays managements [5]. The diagnosis of intussusception is based on clinical history and examination [9], however supportive investigations such us abdominal ultrasound play a great role for about 97–100 %% accuracy, electrolyte, CBC and C-Reactive protein all used to assess patient hemodynamic status [7].

The recommended mode of management are mainly depend on hemodynamic stability of patient [10], but basically it can either be nan-surgical (i.e. pneumatic pressure, hydrostatic enema/saline) or surgical (laparotomy or laparoscopic) [6].

In this case, we presented a rare case of ileal caecum intussusception presenting as a rectal prolapse for a 4-month-old female baby, this was diagnosed after a high suspicious from the history, clinical examination and abdominal ultrasound result. It was then managed successfully by emergency laparotomy and ilea-transverse end to end anastomosis as recommended in the other literatures [11].

Ilea caecum intussusception is the commonest type of intussusception in children contributing for about 90 % of all intussusception cases [2], but with intussusceptum advancement to the anus is often very rare and in case are usually easily misdiagnosed and mismanaged [9], missed intussusception led to delayed management and worsen patient's prognosis [7].

Early intussusception detection is essential to prevent treatment setbacks and possible consequences, this case highlights the significance of a comprehensive clinical assessment and suitable imaging investigations in infants exhibiting symptoms related to the rectum.

## 3 Conclusion

Diagnosing infants with rectal complaints can be difficult and even in cases where clinical signs first point to rectal prolapse. Therefore, a condition like ileocecal intussusception of the colon should be included in the differential diagnosis.

## Consent

Informed consent was obtained from the patient's parent for publication and any accompanying images. A copy of written consent is available for review by the Editor-In-Chief of this journal on request.

## Ethical approval

N/A.

## Funding

N/A.

## Author contribution

1. Fred lazier; main and Corresponding author from developing the title to the writing the entire research
2. Frank Joachim: participated on management of the patient and follow-up
3. Antony Lobulu: participated on clacking a patient and compilation

## Guarantor

Medical officer in charge Mt. Meru regional hospital.

## Research registration number

1. Name of the registry: the work has been reported inline with the SCARE criteria.
2. Unique identifying number or registration ID: N/A.
3. Hyperlink to your specific registration (must be publicly accessible and will be checked):

## Conflict of interest statement

N/A.
